# Editorial: Towards a new 3Rs era in experimental research

**DOI:** 10.3389/fnbeh.2024.1404294

**Published:** 2024-04-15

**Authors:** Christopher R. Cederroth, Jenny Sandström

**Affiliations:** Swiss 3R Competence Center, Bern, Switzerland

**Keywords:** 3Rs, replace, reduce, refine, ethics, animal welfare

The last decade has seen a rise in the interest to apply the 3Rs principle in pre-clinical research. This 64 years old principle, formulated by Russell and Burch in 1959, has been incorporated in many animal welfare legislations to make research more responsible, more ethical and of better quality. Being at the crossroad of innovation, implementation, ethics and society, the advancement of 3Rs has never been more critical (Grimm et al.) and has been the focus of recent international networking initiatives,^1^ funding incentives,^2^ and the creation of 3R centers across Europe (Neuhaus et al., 2022a,b).

**The aim of this Research Topic** is to provide an inter- and multi-disciplinary view on the most advanced level of 3Rs research. This includes a broad range of research foci spanning from human disease models, 3D culture systems, organoid models, non-sentient invertebrates, computational modeling, imaging, animal welfare, education, and legislation. Furthermore, it shows that the coordination of multiple scientific disciplines including biomedical, veterinary, biostatistics, biotechnology, and computer sciences is required to tackle the future challenges for 3Rs. We also know that research alone will not suffice, but that educational, social, political, and ethical perspectives are crucial to shape 3Rs research and its implementation. Within this topic current knowledge on ***replacement***, ***reduction*, **and ***refinement*** is reviewed, spanning 14 associated journals and attesting to the requirement for trans-and cross disciplinarity of 3Rs research to keep at pace with advancing 3Rs methodologies for ethical and humane research practices in the rapidly evolving fields of life sciences, where the 3Rs Principles remain at its core.

**Statistics on this Research Topic:** This Research Topic was open between the 29th of November 2022 and the 28th of February 2023. It received 37 submissions by 231 authors, of which 36 were finally accepted after a peer reviewing process and the topic has to date received over 207,000 views (20^th^ of March 2024).

**Overview of this Research Topic**: This Frontiers Research Topic commences with three Frontiers Young Minds articles, which form part of a broader educational initiative by the Swiss 3Rs Competence Centre aimed at providing an overview of each of the 3Rs for the general public (Hartung; Tremoleda; Jirkof). Following these, Tappe et al. present a hypothesis on assessing post-operative severity in rodents. The first chapter focuses on **replacement**, exploring the validity of disease models in cellular systems and non-sentient invertebrates. The second chapter delves into **reduction** and the long-term monitoring of animals, while the third chapter discusses **refinement** and various methodologies for assessing animal welfare. Concluding the topic are two **perspectives on animal welfare**. It's worth noting that some articles may overlap between replacement and reduction and there remains ongoing debate about whether organisms like zebrafish and fruit flies can truly replace rodents. Ethically, this debate hinges on whether these organisms are considered sentient beings, which can vary depending on legislation and perspective.

## 1 Replacement

Replacement are “*methods, which permit a given purpose to be achieved without conducting experiments or other scientific procedures on animals*.” In this Research Topic, Zhou et al. provide a review on how organoid biobanks may help in drug discovery and precision medicine. Other reviews covered the advancements in 3D cell culture to model tumors and their microenvironment (Zhang et al.), accompanied by a bibliometric analysis of tumor organoid research (Shuoxin et al.). Research on pulmonary or liver diseases using organoids is also reviewed (Bosákov et al.; Liu et al.), and the challenges with brain organoids are discussed (Passaro and Stice). New organoids for replacing sheep in research on ruminant host-pathogen interactions are proposed (Smith et al.). Several articles present the fruit fly *Drosophila melanogaster* as an emerging model for studying exercise and aging (Ding et al.), nociception (He et al.), and Huntington's Disease (Chongtham et al.). In the same direction, the embryons of the zefrafish *Danio rerio* were suggested as models of nodavirus infection (Lama et al.), systemic inflammation (Sebo et al.), and cancer gene therapy (Cascallar et al.). Finally, and to our knowledge a first, tumor organoids derived from a patient with a gastrointestinal stromal tumor mimicked the patient's positive response to sunitinib, suggesting that patient-derived organoids may help predicting responses to drug interventions against cancer (Cao et al.).

## 2 Reduction

Reduction are “*methods for obtaining comparable levels of information from the use of fewer animals in scientific procedures, or for obtaining more information from the same number of animals*.” In this regard, technologies have made significant advances for instance in the monitoring of zebrafish behavior and health (Magalhães et al.; Vossen et al.). New methods to optimize metabolomic assays across various model organisms (e.g., mouse, fruit fly and zebrafish) were proposed by Gegner et al.. In a mouse model of diabetes, the transplantation of human-derived pancreatic islet organoids were labeled and tracked with magnetic particle imaging using mCT, enabling to track the progress of the transplant *in vivo* over a month (Sun et al.). Finally, a large systematic review covered animal and human evidence to demonstrate the benefits of using neuroimaging in pre-clinical models of amyotrophic lateral sclerosis (Cannon et al.).

## 3 Refinement

Refinement encompasses *methods aimed at alleviating or minimizing potential pain, suffering, and distress while enhancing animal wellbeing*. Several articles in this area focus on improving welfare comparisons among different non-human species (Gaffney et al.) and enhancing transparency in laboratory animal science(Enkelmann and Bischoff). Additionally, two studies advocate for the use of facial expressions to monitor animal pain or distress (Fischer-Tenhagen et al.; Swan et al.). The enrichment loss hypothesis posits that the deprivation of enrichment may lead to stress and anxiety. Interestingly, the touchscreen method, designed to reduce the impact of experimenters on animals, did not induce stress effects upon termination of training (Quante et al.). However, the authors urge caution and call for further research to evaluate the benefits and potential negative effects of touchscreen technology. Moreover, glucocorticoids have often been utilized as a surrogate marker for stress and animal welfare. Tiemann et al. conducted a systematic review and concluded that evidence supporting such a correlation is still lacking. In addressing this challenge, Talbot et al. developed RELSA (RELative Severity Assessment), a tool integrating various outcome measures to assess severity and welfare impact. Furthermore, environmental enrichment has garnered significant attention for refining laboratory animal procedures. Mieske et al. employed a systematic review approach to highlight the benefits of a stimulating environment on animal welfare. Here again, the assessment of animal welfare remains a topic of debate, with varying methodologies across studies, primarily relying on behavioral assessments.

## 4 Perspectives

Concluding this Research Topic, Smith introduces Norecopa, a website summarizing the steps in preparing animal studies, primarily based on the PREPARE guidelines, aimed at enhancing planning and reducing animal use in research.

## 5 Concluding remarks

This Research Topic explores interdisciplinary facets of the latest 3Rs research, covering advancement in replacement with 3D culture systems, organoids, and non-sentient invertebrates, as well as cutting-edge imaging techniques, impact reduction and refine animal procedures for better welfare.

The role of innovation in driving progress within the realm of 3Rs cannot be overstated. From pioneering techniques like organoids and on-a-chip systems, to sophisticated new approaches to assess animal wellbeing, each representing the vast range where technological innovation and advancement come to practical use in a 3Rs context. However, while these advancements showcase the potential of scientific innovation to revolutionize research practices, their true impact is contingent upon effective implementation and adoption across the scientific community.

In conclusion, this Research Topic attests to the multitude of disciplines that are involved in pushing the frontiers of 3Rs research forward and points to some of the challenges we are facing in better defining indicators for measuring successful implementation of 3Rs methodologies that can inform the pursuit of the most impactful 3Rs research. It is also an acknowledgment to the robust engagement by the research community to advance 3Rs research and tackle the multidisciplinarity challenges that 3Rs research and implementation entail.

## Author contributions

CC: Conceptualization, Project administration, Writing – original draft, Writing – review & editing. JS: Funding acquisition, Resources, Writing – original draft, Writing – review & editing.

## Footnotes

^1^ https://cost-improve.eu/

^2^ https://www.nfp79.ch/en
