# Erratum: Editorial: Towards a new 3Rs era in experimental research

**DOI:** 10.3389/fnbeh.2024.1434372

**Published:** 2024-06-04

**Authors:** Frontiers Production Office

**Affiliations:** Frontiers Media SA, Lausanne, Switzerland

**Keywords:** 3Rs, replace, reduce, refine, ethics, animal welfare

Due to a production error, [Düpjan, S., and Dawkins, M., S. (2022). Animal Welfare and Resistance to Disease: Interaction of Affective States and the Immune System. *Front Vet Sci*. 9:929805. doi: 10.3389/fvets.2022.929805 was cited in the article as a Perspective that was part of the Research Topic **[Towards a New 3Rs Era in Experimental Research]**, which was incorrect.

A correction has been made in section **4 Perspectives:**

“Concluding this Research Topic, Smith introduces Norecopa, a website summarizing the steps in preparing animal studies, primarily based on the PREPARE guidelines, aimed at enhancing planning and reducing animal use in research.”

The publisher apologizes for this mistake. The original article has been updated.

